# Effects of a Sorghum Beverage with *Lacticaseibacillus paracasei* on Body Composition, Lipid Profiles, and Intestinal Health in Overweight and Obese Adults: A Randomized Single-Blind Pilot Study

**DOI:** 10.3390/foods13193128

**Published:** 2024-09-30

**Authors:** Lucimar Aguiar da Silva, Vinícius Parzanini Brilhante de São José, Larissa Arruda Rodrigues, Pietra Vidal Cardoso do Prado, Renata Celi Lopes Toledo, Frederico Augusto Ribeiro de Barros, Andressa Moreira de Souza, Rosemar Antoniassi, Carlos Wanderlei Piler de Carvalho, Valéria Aparecida Vieira Queiroz, Karina Maria Olbrich dos Santos, Joseph Francis Pierre, Bárbara Pereira da Silva, Hércia Stampini Duarte Martino

**Affiliations:** 1 Department of Nutrition and Health, Federal University of Viçosa, Purdue Avenue, s/n, University Campus, Viçosa 36570-900, MG, Brazil; lucimar.aguiar@ufv.br (L.A.d.S.); vinicius.sao@ufv.br (V.P.B.d.S.J.); larissa.rodrigues1@ufv.br (L.A.R.); pietra.prado@ufv.br (P.V.C.d.P.); renatacelly@yahoo.com.br (R.C.L.T.); barbara.p.silva@ufv.br (B.P.d.S.); 2 Department of Food Technology, Federal University of Viçosa, Purdue Avenue, s/n, University Campus, Viçosa 36570-900, MG, Brazil; fredbarros@ufv.br; 3 Embrapa Agroindústria de Alimentos, Avenida das Américas, 29501, Guaratiba, Rio de Janeiro 23020-470, RJ, Brazil; andressa.moreira@embrapa.br (A.M.d.S.); rosemar.antoniassi@embrapa.br (R.A.); carlos.piler@embrapa.br (C.W.P.d.C.); karina.dos-santos@embrapa.br (K.M.O.d.S.); 4 Embrapa Milho e Sorgo, Br 424, Km 45, Zona Rural, Sete Lagoas 35701-970, MG, Brazil; valeria.vieira@embrapa.br; 5 Department of Nutritional Sciences, University of Wisconsin-Madison, 1415 Linden Dr., Room 340B, Madison, WI 53706-1571, USA; jpierre@wisc.edu

**Keywords:** probiotic, SCFA, intestinal health, Castelli index, visceral fat, *Sorghum bicolor* (L.) Moench

## Abstract

(1) Background: This study aimed to evaluate the effect of an extruded whole-grain sorghum beverage containing *L. paracasei* on body composition, lipid profiles, and intestinal health in overweight and obese adults. (2) Methods: A chronic, single-blind randomized controlled pilot study was conducted with 30 volunteers allocated to three groups (*n* = 10/group): extruded sorghum beverage (ESB), extruded sorghum beverage with *L. paracasei* (ESPB), and control beverage (CB) (waxy maize starch). The chemical composition of the beverages was analyzed. Volunteers consumed the beverages for ten weeks at breakfast, along with individual dietary prescriptions. Body composition, biochemical markers, gastrointestinal symptoms, stool consistency, intestinal permeability, short-chain fatty acids, fecal pH, and stool *L. paracasei* DNA concentration were analyzed at the beginning and end of the intervention period. (3) Results: The ESB showed better composition than the CB, particularly in terms of resistant starch content, total phenolic compounds, condensed tannins, and antioxidant capacity. Both the ESB and the ESPB had an effect on body composition (estimated total visceral fat and waist volume), biochemical markers (Castelli index I), and intestinal health (Bristol scale, diarrhea score, valeric acid, and *L. paracasei* DNA concentration). No changes were observed in the CB group after the intervention. (4) Conclusions: Whole-grain sorghum beverages demonstrated good nutritional value, and consumption of these beverages, with or without *L. paracasei*, provided health benefits, including improvements in body composition, Castelli index I scores, and intestinal health, in overweight and obese adults.

## 1. Introduction

Sorghum (*Sorghum bicolor* (L.) Moench) is the fifth most produced cereal in the world and a staple crop in the diet of more than 750 million people [1,2]. Among the various sorghum genotypes, the hybrid BRS 305 stands out for its high content of resistant starch and bioactive compounds, such as condensed tannins and other phenolic compounds [3,4,5]. Despite this, in Brazil, most sorghum is used for animal feed, with only 4% being used for human consumption, seeds, and industry [6].

Thus, the development of new products using this food matrix and the use of different processing methods may be efficient strategies to increase the consumption of this cereal. Among these processes, extrusion cooking combines heat, high pressure, and mechanical shear, which are capable of altering the structure of low-water-content whole-grain sorghum, a product consisting largely of starch, insoluble fiber, and proteins, making these components more soluble and enabling the development of sorghum beverages. Furthermore, this process has been proven to effectively increase the availability of nutrients such as phenolic compounds, including condensed tannins [3,7], and to improve body composition, promote weight loss, and enhance intestinal health in overweight adult men [8,9].

The health benefits of dietary fiber and phenolic compounds can be enhanced when they are combined with probiotics [10]. Strains of *Lacticaseibacillus* spp. have been shown to exhibit probiotic properties, particularly within the species *Lacticaseibacillus paracasei* and *Lacticaseibacillus rhamnosus*. *L. paracasei* is commonly found in milk and cheese, and in vitro tests have demonstrated its beneficial properties and safety as a promising probiotic that can be added to products due to its high resistance to simulated gastrointestinal conditions [11].

Studies have shown that strains of *L. paracasei* reduce weight gain and body adiposity and improve lipid profiles in obesity-induced mice [12,13,14]. This probiotic also improved intestinal morphology by increasing the height and depth of villi and crypts and the number of Paneth cells and restoring intestinal microbiota profiles in *Gallus gallus* models [15]. Furthermore, doses ranging from 10⁶ to 10^1^⁰ CFU of *L. paracasei* increased short-chain fatty acid production in healthy adults [16] and improved lipid profiles, intestinal barrier function, and intestinal permeability in elderly people [17].

We have not found any studies investigating the health effects of probiotic microorganisms associated with plant-based foods or chronic studies using unfermented beverages prepared with extruded sorghum flour. Since the starch in extruded flour has a high degree of solubilization, this flour is suitable for preparing drinks, soups, and other foods without heating. A fermented sorghum-based beverage increased antioxidant activity and improved the lipid profile in rats fed a high-fat diet [18]. The consumption of a beverage containing extruded sorghum reduced the glycemic response to a subsequent meal in an acute study with normal-weight adults [19].

This research evaluated the effect of *L. paracasei* supplementation in plant products without fermentation. Therefore, the combined use of extruded sorghum and *L. paracasei* offers an option to include probiotics in plant-based diets, which may benefit overweight and obese individuals, as these populations often experience metabolic alterations such as dyslipidemia and intestinal dysbiosis [20]; these probiotics may also reduce constipation [21]. Additionally, this diet meets the dietary needs of vegans, lactose-intolerant individuals, and those with allergies to animal proteins. Thus, this study aimed to evaluate the effect of an extruded whole-grain sorghum beverage associated with *L. paracasei* TRA061676 on the body composition, lipid profile, and intestinal health of adults with overweight and obesity.

## 2. Materials and Methods

### 2.1. Beverage Preparation

BRS 305 sorghum grain was produced and donated by Embrapa Milho e Sorgo (Sete Lagoas, Minas Gerais, Brazil), and *Lacticaseibacillus paracasei* TRA061676 was cultivated and purified at Embrapa Agroindústria de Alimentos (Rio de Janeiro, RJ, Brazil). The extrusion cooking process was performed using a co-rotating twin-screw extruder, the Evolum HT25 (Clextral Inc., Firminy, France), with a 40:1 length/diameter ratio and ten heating zones. The flour was fed into the extruder’s feed zone using a loss-in-weight gravimetric feeder, the GRMD15 (Schenck Process, Darmstadt, Germany), monitored by the Schenck Process Easy Serve software system version 15.8 (Schenck Process, Darmstadt, Germany), at a constant feed rate of 10 kg/h, with the temperature of the last zone maintained at 130 °C. Deionized water was injected between the first and second modular zones using a Super K PP 6.35 plunger metering pump (DKM Clextral Inc., Firminy, France) to achieve a moisture content of 14%. The extrudates were cut with a four-blade knife installed at the die exit to produce small extrudates, which were dried in a fan oven at 60 °C for 2 h and then ground in a hammer mill fitted with a 1 mm opening to obtain a fine flour (90% passing through a 150 µm opening).

The extruded sorghum beverage (ESB) was developed using extruded sorghum flour (11.54%), maltodextrin (11.54%), and soy milk (38.46%), and flavored with 100% whole juice in various flavors (apple, mango, guava, grape, and peach) (38.46%). For the extruded sorghum beverage with probiotics (ESPB), 0.2 g of freeze-dried *Lacticaseibacillus paracasei* TRA061676 [22] was added (1.08 × 10⁹ CFU/g) to the ESB. This probiotic was isolated from *coalho* cheese and cultivated in extruded sorghum flour, making it the first probiotic completely isolated and produced in Brazil. The control beverage (CB) had the same composition as the test beverages, except that the sorghum flour was replaced with waxy maize starch. The final volume of the beverage was approximately 250 mL for women and 340 mL for men. The portion size was chosen to replace the total breakfast calories (15% of the daily caloric requirement based on a 2000-calorie diet for women or a 2500-calorie diet for men). Beverages were provided to volunteers weekly and stored under refrigeration, with instructions to consume one bottle daily at breakfast. At the end of each week, volunteers returned the empty bottles and a daily consumption log.

### 2.2. Chemical Composition of Beverages

The chemical composition of sorghum and control beverages was analyzed. Moisture was determined by oven drying at 105 °C (Nova Ética, São Paulo, Brazil). Protein content was assessed using the micro-Kjeldahl method. Total dietary fiber and insoluble and soluble fractions were quantified by a gravimetric–enzymatic method using a total dietary fiber kit (Megazyme^®^, Bray, Irlanda). Total lipid content was determined by the Soxhlet method [23]. Carbohydrate content was calculated by subtracting the sum of lipids, proteins, total dietary fiber, moisture, and ash from 100%. The total energy value of the beverages was estimated based on conversion factors of 4 kcal/g for protein and carbohydrate and 9 kcal/g for lipids [24]. Resistant starch content was quantified with a K-RSTAR enzyme kit (Megazyme^®^, Bray, Irlanda). Total phenolic content was analyzed using the Folin–Ciocalteu method. The result was expressed as milligrams of gallic acid equivalents per gram of the sample (mg GAE/g). Condensed tannin content was analyzed using the vanillin/HCl reaction method, according to Maxson and Rooney [25] and Price, Van Scoyoc, and Butler [26]. Condensed tannin content was expressed as milligrams of catechin equivalents per gram of sample. Antioxidant activity was evaluated using a reaction with DPPH (1,1-diphenyl-2-picrylhydrazyl). The antioxidant activity was expressed in µmol of Trolox equivalent per gram of the sample (µmol Trolox/g) [27].

### 2.3. Study Design

**Figure 1:**
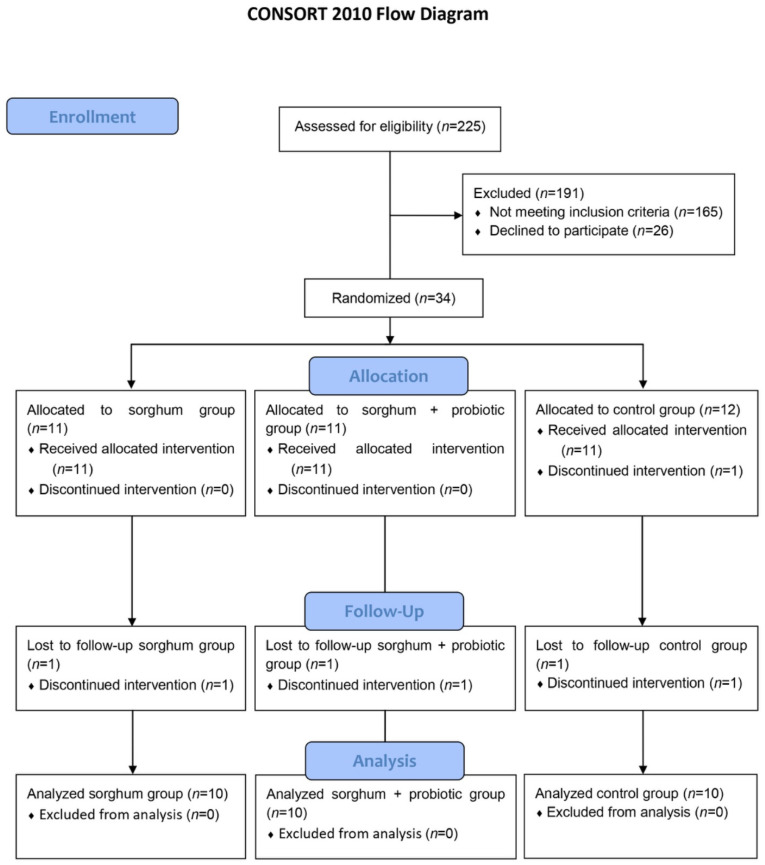
CONSORT flow diagram outlining the design and conduct of the clinical study.

A single-blind, randomized, controlled, parallel pilot study was performed at the Department of Nutrition and Health of the Federal University of Viçosa (UFV), Brazil, between April and December 2023. A total of 30 volunteers were randomized and allocated to three experimental groups by researchers: extruded sorghum beverage (ESB) (*n* = 10), extruded sorghum beverage supplemented with *Lacticaseibacillus paracasei* (ESPB) (*n* = 10), and control beverage (CB) (*n* = 10). The sample size was determined according to Julious (2005) [28] (Figure 1). The allocation was performed using the MinimPy software, version 2.0 (Copyright, Mahmoud Saghaei, 2011), and the variables sex, age, and body mass index were used to balance the potential factors that could interfere with the outcome variables. Volunteers received a nutritional intervention with a calorie restriction of 500 kcal/day and drank their respective breakfast beverages for ten weeks. Volunteers did not know which beverage they were receiving. Volunteers visited the laboratory at the beginning and end of the intervention for body composition assessment, blood collection, and completion of the gastrointestinal symptoms questionnaire and the Bristol scale. After 4.5 h of collection from the permeability test, each volunteer provided urine and fecal material, which was collected and kept refrigerated until delivery.

All procedures performed in this study involving humans followed the ethical standards of the Federal University of Viçosa. The study protocol was approved by the Human Research Ethics Committee of the Federal University of Viçosa (number 5.162.838/CAAE: 53827321.4.0000.5153—24 November 2021) and registered in the Brazilian Registry of Clinical Trials (registration number: RBR-32v2gm5).

### 2.4. Study Population

Adult volunteers (both sexes) between 20 and 55 years old with a body mass index (BMI) between 27.0 and 34.9, a body fat percentage above 30% for female and 20% for males, a waist circumference above 80 cm for females and 92 cm for males, and a light physical activity level (less than 150 min/week) [29] were included in the study. The exclusion criteria were as follows: alcoholism; smoking; pregnancy or lactation; history of recent digestive, hepatic, renal, cardiovascular, thyroid, or inflammatory diseases; continuous anti-inflammatory drug and/or corticosteroid intake, or laxative or antibiotic intake within three months prior to the study; use of probiotic, prebiotic, or synbiotic products more than twice a week in the month prior to the study; recent physical activity level change; and eating disorders [30].

### 2.5. Body Composition

Body composition was assessed by dual-energy X-ray absorptiometry (DEXA) (Lunar Prodigy Advance DXA System, version 13.31, GE Lunar), following the manufacturer’s recommendations, where weight, body fat percentage, lean mass, estimated total visceral fat (TVF), and abdominal volume were evaluated. Height was measured using a vertical anthropometer (Alturexata Ltd.a., Belo Horizonte, Brazil), and body mass index (BMI) was calculated (weight/height^2^).

### 2.6. Biochemical Markers

Venous blood samples were collected after a 12 h overnight fast. The blood was centrifuged at 2422 g for 15 min at 4 °C, and total cholesterol, high-density lipoprotein cholesterol (HDL-c), low-density lipoprotein cholesterol (LDL-c), triglyceride, and glucose concentrations were determined using commercial kits (Bioclin^®^ Company, Belo Horizonte, Brazil) according to manufacturer instructions. Insulin and ultra-sensitive C-reactive protein (CRP) were measured in serum using chemiluminescence and immunoturbidimetry methods in a third-party laboratory. Castelli index I (total cholesterol/HDL-c) and Castelli index II (LDL-c/HDL-c) were calculated.

### 2.7. Gastrointestinal Symptom Rating Scale and Bristol Stool Form Scale Questionnaire

Volunteers answered a gastrointestinal symptom rating scale (GSRS) questionnaire [31] comprising 15 questions combined into five groups of symptoms representing reflux, abdominal pain, indigestion, diarrhea, and constipation. The GSRS has a seven-point graduated Likert scale where 1 represents no bothersome gastrointestinal symptoms and 7 represents very bothersome symptoms in the last week. The GSRS score (average of all five sub-scores) was calculated. The Bristol stool form scale questionnaire [32] was applied to obtain information about intestinal transit and functionality.

### 2.8. Intestinal Permeability Test

Intestinal permeability was assessed by quantifying urinary excretion of lactulose and mannitol. After overnight fasting, volunteers drank a test solution containing 250 mL of water, 10 g of lactulose, and 5 g of D-mannitol. Two and three hours after the test started, 150 mL of water was offered, and all excreted urine was collected until 4 h 30 min. The total volume of excreted urine was measured, and thimerosal (4:1, mg: mL) was added, after which the samples were stored at −20 °C until analysis.

High-performance liquid chromatography (HPLC) was used to determine the lactulose and mannitol concentrations. The collected urine was filtered through 0.22 mm Millipore filters, and approximately 1.5 mL was placed in HPLC vials. The analyses were performed with a Bio-Rad HPX 87H column, 300 × 7.8 mm, maintained at 45 °C using a Shimadzu Prominence LC-20A chromatograph (Shimadzu, Milan, Italy) coupled to a Shimadzu 20A refractive index detector (RID). The mobile phase was 5.0 mM sulfuric acid (H_2_SO_4_) with a 0.7 mL/min flow rate. Known amounts of lactulose (12.5 to 0.1953 mM) and mannitol (25 to 0.3906 mM) were used as standards. The lactulose and mannitol excretion rates were calculated using the formulas [(urinary mannitol x urinary volume excreted)/5 g mannitol ingested] × 100 and [(urinary lactulose × urinary volume excreted)/10 g lactulose ingested] × 100, and the lactulose/mannitol ratio was calculated.

### 2.9. Short-Chain Fatty Acid Analysis

Stool samples were weighed (200 milligrams) and dissolved in 2 mL of water. The extraction of SCFAs was performed with ethyl ether using a vortex followed by centrifugation. The analysis was carried out by gas chromatography on Agilent 7890A (Wilmington, DE, USA) equipped with a flame ionization detector and fitted with an FFAP capillary column (nitroterephthalic acid-modified polyethylene glycol, 25 m × 0.2 mm × 0.30 μm). The temperature was programmed to increase from 40 to 230 °C, and the injector and detector were kept at 250 and 280 °C, respectively. The identification was carried out with standards from Sigma-Aldrich (St. Louis, MO, USA). For quantification, calibration curves were used based on the ratio of concentration and ratio of area between the internal standard (crotonic acid) and acetic, propionic, butyric, isobutyric, valeric, isovaleric, and hexanoic acids. The results are expressed in mmol of fatty acid per kg of feces.

### 2.10. Fecal pH

Fecal pH was measured using a digital pH meter (Instrutherm^®^, model PH-1900) after diluting 100 milligrams of fecal samples in deionized water (pH = 6.62) at a concentration of 100 mg/mL [33].

### 2.11. DNA Extraction and Fecal PCR Analysis

DNA extraction was performed using the QIAamp PowerFecal Pro DNA Kit (Qiagen^®^, Hilden, Germany) according to the manufacturer’s instructions. The flow-through, with DNA, was collected and stored at −80 °C until analysis.

The *Lacticaseibacillus paracasei* concentration was measured using a SYBR Green detection system (Molecular Probes, Inc., Eugene, OR, USA). The polymerase chain reaction (PCR) primer sequences were as previously described: forward, 5-GCACCGAGATTCAACATGG-3, and reverse, 5-GGTTCTTGGATYTATGCGGTATT-3 [34]. The thermocycling parameters for conducting real-time PCR were defined as polymerase activation (95 °C/15 min), denaturation (95 °C/15 s), and annealing (60 °C/min) for 40 cycles (Applied Biosystems 7500 Real-Time PCR). To construct a *Lacticaseibacillus paracasei* standard curve, DNA from an isolated colony of *L. paracasei* derived from a single ancestor was compared with a positive control (PC) *L. paracasei* sample for subsequent quantification. Standard and PC DNA were extracted with a ZymoBiomics D4300 kit (Zymo Research^®^, Orange, CA, USA) and quantified on a Nanodrop™ One^C^ (ND-ONEC-W, Thermo Fisher Scientific Inc., Waltham, MA, USA). Quantification results were expressed as ng/µL of *L. paracasei* species DNA concentration.

### 2.12. Statistical Analysis

The statistical analysis was performed in GraphPad Prism version 9.0.2, adopting a *p*-value of <0.05 as significance, and trend-level significance was defined by a *p*-value < 0.1 [35]. The Shapiro–Wilk test was performed to verify each variable’s normality, and the data are presented as means and standard deviations, bar graphs, and box-and-whisker plots. One-way ANOVA followed by the Newman–Keuls test or the Kruskal–Wallis test followed by Dunn’s test was used to compare the groups.

## 3. Results

### 3.1. Participants

Two hundred twenty-five individuals expressed interest in participating in the study. The following were not included: eighty-two normal-weight individuals, forty individuals with a body mass index above 35, one with a body fat percentage below the minimum stipulated for the study, fourteen who performed more vigorous physical activity or who had changed their level of physical activity recently at the beginning of the study, one individual over 50 years old, five who had used antibiotics less than three months before the beginning of the study, seventeen who had a disease or took a medication that altered some parameter of the study, two who smoked, one who was pregnant, one scheduled for elective surgery, one who consumed probiotics daily, and 26 who withdrew from the study or did not respond to the contact form. Thus, 34 individuals were selected and randomized: 11 individuals for the EPSB, 11 for the ESB, and 12 for the CB. After the start of the study, one volunteer from the sorghum group was excluded because he needed to start antibiotic therapy; one from the sorghum + probiotic group and two from the control group were excluded because they no longer wished to participate. Thus, 30 individuals completed all stages of the study: 10 in the sorghum group, 10 in the sorghum + probiotic group, and 10 in the control group (Figure 1). The two groups presented similar age (ESPB 33.4 ± 7.0, ESB 35.00 ± 8.4, and CB 36.1 ± 10.3 years old; *p* = 0.784) and gender distributions (ESPB eight women/two men, ESB: eight women/t men, and CB eight women/two men). The three groups presented similar BMIs at the start of the intervention (ESB 29.09 ± 2.37, ESPB 29.77 ± 2.83, and CB 29.88 ± 2.50 kg/m^2^; *p* = 0.727).

### 3.2. Beverage Chemical Composition

Among the macronutrients, the ESB presented a higher content of moisture, protein, total dietary fiber, soluble dietary fiber, and insoluble dietary fiber and a lower content of carbohydrates, ash, and energy than CB. Although there were statistically significant differences in moisture, carbohydrates, and ash, the values differed only slightly. No difference was observed between the beverages regarding lipid content or energy density. Regarding bioactive compounds and antioxidant capacity analysis, the ESB presented a higher content of resistant starch, total phenolic compounds, condensed tannins, and antioxidant capacity than the CB, and these characteristics increase the potential for sorghum to provide health benefits. The ESPB had the same composition as the ESB, except for the addition of *Lacticaseibacillus paracasei*, which did not influence the parameters evaluated (Table 1).

### 3.3. Effects of ESB with Lacticaseibacillus paracasei TRA061676 on Body Composition and Biochemical Markers

Analysis of the delta of estimated total visceral fat (ΔTVF) from baseline to the endpoint of the study revealed that the ESPB and ESB groups had different values of ΔTVF compared to the CB group. The negative delta in the ESPB and ESB groups means that the values found at the endpoint for TVF were lower than those at baseline in those groups. In the CB group, the ΔTVF was positive, meaning that the values found at the endpoint for TVF were higher than those at baseline. Similarly, analysis of the delta of estimated waist volume (ΔWV) from baseline to the end of the study endpoint revealed that the ESB group had different values of ΔWV compared to the ESPB and CB groups. However, no difference was observed among groups for body weight, BMI, body fat, lean mass, estimated visceral adipose tissue, or waist volume at baseline or at the endpoint, nor the delta of any of the values (Table 2).

Castelli index I (total cholesterol/HDL-c) was reduced in the ESPB and ESB groups after ten weeks of intervention (endpoint) (Table 3). The ESB group showed a trend (*p* = 0.089) toward reduced CRP compared to the ESPB and CB groups at the endpoint. However, no difference among the groups was observed for the glucose, insulin, total cholesterol, HDL-c, LDL-c, or triglyceride concentration or Castelli index II (LDL-c/HDL-c).

### 3.4. Effects of EBS in Combination with Lacticaseibacillus paracasei TRA061676 on Intestinal Health

**Figure 2:**
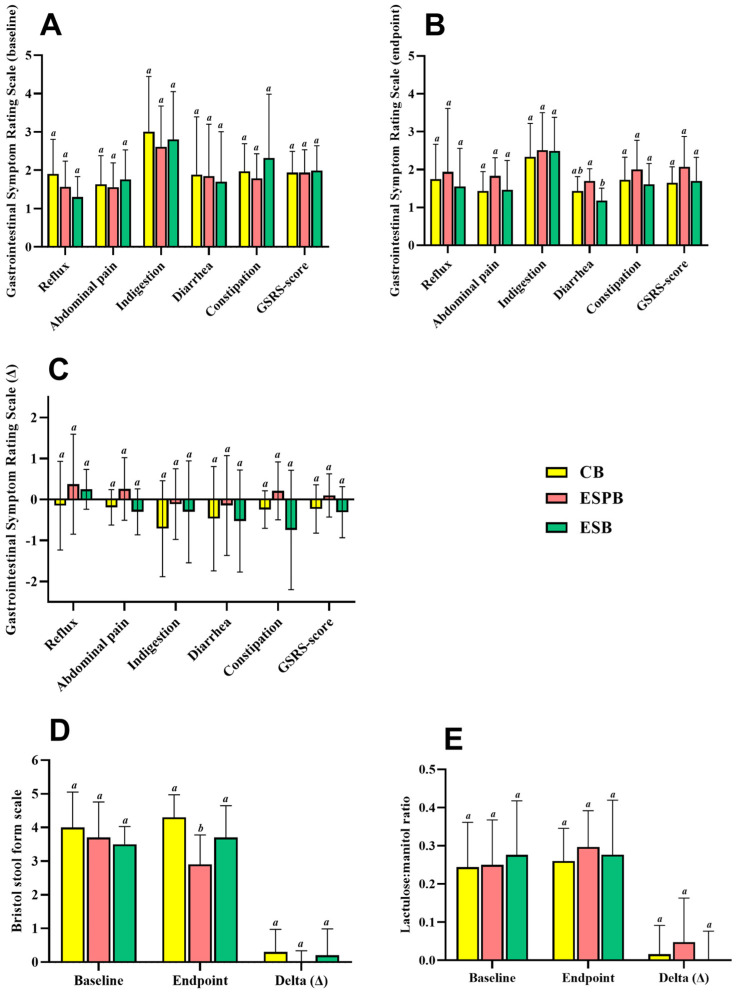
Gastrointestinal symptoms of overweight and obese adults after consuming beverages supplemented or not with a *Lacticaseibacillus paracasei* TRA061676 probiotic—gastrointestinal symptom rating scale (GSRS) baseline (**A**), endpoint (**B**), and delta (endpoint—baseline assessment) (**C**) scores based on severity of symptoms; stool consistency measured by the Bristol scale (**D**); intestinal permeability (**E**). ESPB: extruded sorghum beverage with an added probiotic; ESB: extruded sorghum beverage; CB: control beverage. Delta (∆): endpoint—baseline assessment. Different letters mean a statistically significant difference between the groups as determined by ANOVA with a post hoc Newman–Keuls test and the Kruskal–Wallis test with a post hoc Dunn’s test. (*p* < 0.05). *n* = 10/group.

There were no differences among groups in gastrointestinal symptoms related to the presence of reflux, abdominal pain, indigestion, constipation, and the GSRS score (Figure 2A–C), although the diarrhea score was higher in the ESPB group than in the ESB group at the endpoint (Figure 2B). Regarding stool consistency, the ESPB group presented a lower score on the Bristol scale than the ESB and CB groups at the endpoint (Figure 2D). No difference was observed in intestinal permeability among the groups at the baseline, at the endpoint, or by delta (Figure 2E).

Analysis of the delta of valeric acid from baseline to the study’s endpoint revealed that the ESPB group produced more valeric acid than the ESB and CB groups. No difference was observed in fecal pH among the groups at the baseline, at the endpoint, or by delta (Table 4).

**Figure 3:**
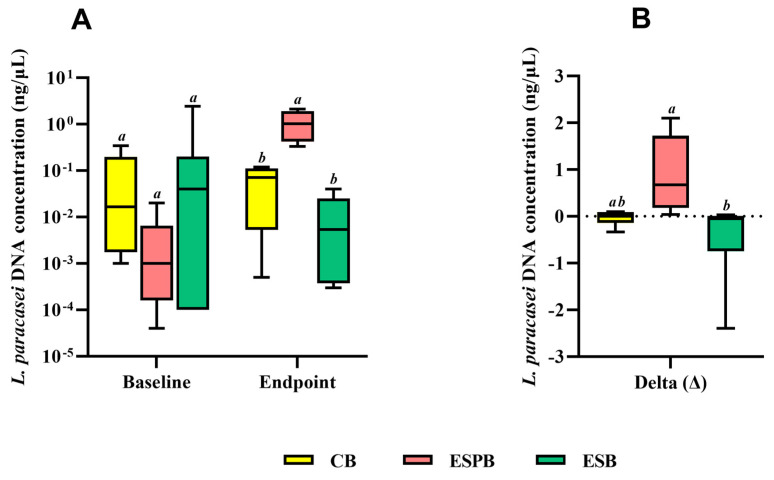
*Lacticaseibacillus paracasei* DNA concentrations before and after intervention with sorghum beverages with and without an added probiotic (**A**), and delta (endpoint—baseline assessment) (**B**). ESPB: extruded sorghum beverage with an added probiotic; ESB: extruded sorghum beverage; CB: control beverage; delta (∆): endpoint—baseline assessment. The horizontal line within each box represents the median; the lower and upper bounds of the box represent the first and third quartiles, respectively; and the lower and upper error bars represent the minimum and maximum values. Different letters mean a statistically significant difference between the groups as determined by an ANOVA with a post hoc Newman–Keuls test (*p* < 0.05). *n* = 10/group.

A difference in the *Lacticaseibacillus paracasei* DNA concentration was observed in the ESPB group compared to the ESB and CB groups at the endpoint (Figure 3A). Analysis of the delta of the *Lacticaseibacillus paracasei* DNA concentration from baseline to the endpoint of the study revealed that the ESPB group had a higher Lacticaseibacillus paracasei DNA concentration than the ESB group (Figure 3B).

## 4. Discussion

This study evaluated the effect of sorghum beverages with or without the addition of Lacticaseibacillus paracasei TRA061676 on the body composition, lipid profiles, and intestinal health of adults with overweight and obesity. ESB provides an elevated nutritional composition; high protein content; dietary fibers; and bioactive compounds, such as resistant starch, phenolic compounds in general, condensed tannins, and antioxidants. BRS 305 sorghum flour is the beverage’s main source of these compounds. These compounds potentially modulate processes related to chronic non-communicable diseases, such as obesity, diabetes, dyslipidemia, and cardiovascular diseases [36]. ESPB resulted in a good matrix to maintain probiotic viability and function, supported by an increased DNA concentration for this bacterium in stool samples, despite only 0.2 g of *Lacticaseibacillus paracasei* being added to the beverage. Accordingly, ESPB increased the valeric acid production and decreased the consistency of feces.

The ESPB and ESB groups showed decreases in Castelli index I. This index is an indicator of cardiovascular disease (CVD) [37], where values higher than 4.3 mg/dL represent a risk of CVD [38]. Since the volunteers in our study are overweight or obese, they have risk factors for the development of CVD, which can be detected by Castelli index I [39]. Although no group presented a Castelli index I score higher than 4.3 mg/dL at the beginning or end of the experiment, the control group was close to this limit at the endpoint. However, the ESPB and the ESB groups presented lower scores than the CB groups. In animal studies, rats fed a normal diet [40] or a high-fat diet (HFD) [41] associated with sorghum showed an increase in HDL-c levels, which can reduce Castelli index I [42,43]. Extruded sorghum, when consumed with or without probiotics, may also have the potential to protect against CVD in individuals with overweight and obesity.

The observed decrease in Castelli index I can be attributed to the high content of phenolic compounds, such as condensed tannins (proanthocyanidins), in the extruded BRS 305 sorghum [44]. In preclinical models, the consumption of sorghum increased the expression of sterol response element binding protein 2 (SREBP2) and ATP-binding cassette subfamily A1 (ABCA1) [42]. The proanthocyanidins are phenolic compounds present in sorghum that have shown the capacity to modulate the activity of SREBP2, which regulates hepatic cholesterol homeostasis via ABCA1 and ATP-Binding Cassette Transporter G1 (ABCG1), increasing HDL-c levels [43].

Furthermore, sorghum consumption reduced estimated total visceral fat and waist volume, which can be attributed to bioactive compounds present in the beverage, such as dietary fiber. The content of this nutrient, including resistant starch, can increase the intestinal transit speed, decreasing fat absorption and increasing saturated fat excretion [45,46]. Decreased fat absorption may be associated with the observed reduction in visceral fat in our ESPB and ESB groups. Similar results were observed in obese rats, where sorghum increased hepatic expression of peroxisome proliferator-activated receptor (PPAR-α) [47], regulating the expression of genes involved in the β-oxidation pathways and fatty acid transport proteins, such as carnitine palmitoyl transferase 1 (CPT-1). These pathways play an important role in fatty acid oxidation and triacylglycerol reduction [47], which may reduce visceral fat and abdominal volume.

Regarding the intestinal parameters, the ESB group showed no change in gastrointestinal symptoms, stool consistency, short-chain fatty acid production, fecal pH, or intestinal permeability. However, when *L. paracasei* was added to the sorghum beverage (ESPB), increased levels of valeric acid production, a reduced score for diarrhea, and improved stool consistency were observed. Valeric acid is a potential therapeutic agent, as it inhibits histone deacetylase (HDAC), which is implicated in the pathogenesis of diseases such as cancer, colitis, and CVD [48]. Valeric acid also improved intestinal barrier integrity [48] in mice fed a high-fat, high-cholesterol diet and exhibited a protective function against non-alcoholic fatty liver disease (NAFLD-HCC) in human cells [49]. Similar results were found in constipated patients: *L. paracasei* increased the production of valeric acid, reduced abdominal distension, and increased bowel evacuation [50].

In cell culture, *L. paracasei* added to sorghum products showed its capacity to use sorghum nutrients as a source of energy, producing SCFAs [51,52]. SCFAs are a source of energy for epithelial cells in the gastrointestinal tract, helping to improve intestinal function, improve stool consistency, reduce gastrointestinal symptoms, and augment mucosal immune function [53]. In our study, this probiotic added to the sorghum beverage also showed intestinal health benefits, increasing valeric acid production and maintaining stool consistency in a normal bowel rhythm. However, the stool consistency score decreased. Although the ESPB group scored below three on the Bristol stool form scale, this score is considered normal bowel rhythm; the intestinal microbiota plays a fundamental role in regulating intestinal motility, affecting transit time and the frequency and consistency of stools. *L. paracasei* mainly acts on overall intestinal peristalsis to relieve constipation, increase intestinal motility, and relieve constipation [54].

Further, the sorghum beverage supplemented with the probiotic increased the *L. paracasei* DNA concentration in the feces, which can correlate with the production of short-chain fatty acids, such as valeric acid. Thus, *Lactobacillus*, such as *L. paracasei*, can increase fermentation, promoting an increase in short-chain fatty acid production, inhibiting the adherence and growth of harmful bacteria and other pathogens, and stimulating colonic blood flow and growth of epithelial cells [55].

Despite the promising results, this is a pilot trial, and some limitations of this study should be considered. The small sample size and the short duration of the intervention are the main limitations, increasing the risk of information bias, and caution is recommended when generalizing the results found. Potential sex differences in the health benefits of ESB and ESPB were not assessed due to the number of participants (eight women and two men in each group). However, these results are encouraging for the replication of this study with a larger, broader sample and a longer duration (at least 12 weeks) to increase the reliability of the data.

## 5. Conclusions

Whole-grain sorghum beverages provide high nutritional value, and their consumption, with or without *L. paracasei* TRA061676, supported health benefits such as improved body composition, Castelli index I, and intestinal health markers in overweight and obese adults. Furthermore, developing non-dairy synbiotic food products without fermentation would result in practical foods that could benefit potential consumers, including vegans, lactose-intolerant individuals, or individuals with milk protein allergies. Future studies are needed to investigate the potential effects on the intestinal microbiota and microbiome.

## Figures and Tables

**Table 1:** Chemical composition of the sorghum beverages and the control.

Variables (g·100 mL^−1^)	ESB	CB	*p*-Value
Moisture	71.07 ± 0.01	70.76 ± 0.19	0.048
Proteins	3.59 ± 0.23	2.49 ± 0.14	0.002
Lipids	0.32 ± 0.09	0.23 ± 0.02	0.102
Ash	0.53 ± 0.00	0.69 ± 0.00	<0.001
Total dietary fiber	0.80 ± 0.25	0.00 ± 0.00	0.010
Soluble dietary fiber	0.04 ± 0.01	0.00 ± 0.00	0.663
Insoluble dietary fiber	0.77 ± 0.15	0.00 ± 0.00	0.025
Carbohydrate	23.71 ± 0.28	25.84 ± 0.05	<0.001
Energy value (kcal·100 g^−1^)	112.08 ± 0.32	115.39 ± 0.13	<0.001
Resistant starch	1.05 ± 0.06	0.24 ± 0.02	<0.001
Total phenolic compounds (mg GAE·g^−1^)	36.37 ± 0.68	4.13 ± 0.08	<0.001
Condensed tannins (mg CE·g^−1^)	0.53 ± 0.03	0.00 ± 0.00	<0.001
DPPH (µmol trolox·g^−1^)	66.64 ± 0.88	0.00 ± 0.00	<0.001

ESB: extruded sorghum beverage; CB: control beverage; GAE: gallic acid equivalents; CE: catechin equivalents; DPPH: 2,2-diphenyl-1-picryl-hydrazyl. Results are expressed as means ± standard deviations. *t* test (*p* < 0.05). *n* = 10/group.

**Table 2:** Body composition of overweight and obese adults at baseline and after consuming sorghum beverages with and without *Lacticaseibacillus paracasei* TRA061676 probiotic, with delta values.

Body Composition	Group			
	ESPB	ESB	CB	*p*-Value
Baseline				
Weight (kg)	75.98 ± 8.98	78.11 ± 13.72	75.4 ± 2.42	0.838
BMI (kg/m^2^)	29.77 ± 2.83	29.09 ± 2.37	29.92 ± 2.54	0.781
Body fat (%)	42.39 ± 7.75	43.8 ± 6.08	45.29 ± 6.67	0.662
Lean mass (kg)	38.18 ± 2.71	39.21 ± 3.24	39.03 ± 6.23	0.897
Estimated TVF (kg)	0.92 ± 0.32	0.95 ± 0.39	0.58 ± 0.51	0.270
Waist volume (m^3^)	0.67 ± 0.36	1.01 ± 0.41	0.61 ± 0.54	0.265
Endpoint				
Weight (kg)	75.82 ± 10.26	77.51 ± 13.97	75.84 ± 2.15	0.927
BMI (kg/m^2^)	29.68 ± 3.20	28.87 ± 2.59	29.88 ± 2.50	0.727
Body fat (%)	40.82 ± 7.30	42.81 ± 5.53	44.83 ± 6.11	0.406
Lean mass (kg)	39.48 ± 3.35	40.05 ± 2.59	39.55 ± 5.29	0.963
Estimated TVF (kg)	0.76 ± 0.30	0.67 ± 0.23	0.66 ± 0.44	0.855
Waist volume (m^3^)	0.63 ± 0.31	0.71 ± 0.24	0.70 ± 0.47	0.889
Delta (Δ)				
Weight (kg)	−0.16 ± 2.5	−0.60 ± 1.45	0.40 ± 1.7	0.615
BMI (kg/m^2^)	−0.07 ± 1.00	−0.22 ± 0.54	−0.04 ± 0.79	0.885
Body fat (%)	−1.57 ± 2.97	−1.06 ± 2.53	−0.46 ± 2.15	0.646
Lean mass (kg)	1.29 ± 2.84	0.84 ± 1.28	0.52 ± 1.92	0.766
Estimated TVF (kg)	−0.15 ± 0.10 ^a^	−0.27 ± 0.23 ^a^	0.08 ± 0.11 ^b^	0.006
Waist volume (m^3^)	−0.04 ± 0.22 ^b^	−0.29 ± 0.25 ^a^	0.09 ± 0.12 ^b^	0.019

ESPB: extruded sorghum beverage with an added probiotic; ESB: extruded sorghum beverage; CB: control beverage; delta (∆): endpoint—baseline assessment; BMI: body mass index; TVF: total visceral fat. Results are expressed as means ± standard deviations. Different letters mean a statistically significant difference between the groups as determined by ANOVA with a post hoc Newman–Keuls test (*p* < 0.05). The *p*-value in the right column is the *p*-value for the ANOVA. *n* = 10/group.

**Table 3:** Biochemical variables in overweight and obese adults at baseline and after consuming sorghum beverages with and without *Lacticaseibacillus paracasei* TRA061676 probiotic, with delta values.

Biochemical Variables	Group			
	ESPB	ESB	CB	*p*-Value
Baseline				
Glucose (mg/dL)	107.1 ± 16.54	99.00 ± 9.45	102.40 ± 16.54	0.575
Insulin (µUI/mL)	6.45 ± 2.69	8.14 ± 2.73	9.66 ± 2.46	0.072
CRP (mg/L)	0.21 ± 0.07	0.09 ± 0.05	0.25 ± 0.19	0.111
Total cholesterol (mg/dL)	155.50 ± 26.38	134.70 ± 13.34	160.00 ± 21.98	0.135
HDL-c (mg/dL)	42.29 ± 7.60	44.89 ± 5.25	49.10 ± 10.53	0.296
LDL-c (mg/dL)	67.61 ± 15.68	62.20 ± 15.32	73.09 ± 13.21	0.404
Triglycerides (mg/dL)	94.32 ± 9.65	90.13 ± 9.04	94.54 ± 5.07	0.432
Castelli index I	3.26 ± 0.83	3.36 ± 0.51	3.81 ± 0.54	0.241
Castelli index II	1.42 ± 0.56	1.30 ± 0.56	1.64 ± 0.46	0.465
Endpoint				
Glucose (mg/dL)	96.00 ± 14.88	98.00 ± 3.26	92.78 ± 6.01	0.548
Insulin (µUI/mL)	6.77 ± 1.98	7.22 ± 2.39	7.56 ± 2.34	0.784
CRP (mg/L)	0.21 ± 0.11	0.09 ± 0.06	0.21 ± 0.11	0.089
Total cholesterol (mg/dL)	140.60 ± 12.51	128.40 ± 27.22	150.70 ± 30.03	0.334
HDL-c (mg/dL)	44.95 ± 6.13	44.64 ± 12.95	44.53 ± 15.02	0.997
LDL-c (mg/dL)	68.09 ± 18.57	56.34 ± 11.20	68.96 ± 10.58	0.217
Triglycerides (mg/dL)	91.94 ± 12.26	87.05 ± 7.93	91.15 ± 11.33	0.580
Castelli index I	3.06 ± 0.34 ^b^	2.54 ± 0.77 ^b^	4.02 ± 1.01 ^a^	0.009
Castelli index II	1.45 ± 0.54	1.12 ± 0.43	1.77 ± 0.64	0.114
Delta (Δ)				
Glucose (mg/dL)	−11.13 ± 14.18	−1.00 ± 9.41	−9.66 ± 15.82	0.326
Insulin (µUI/mL)	0.32 ± 1.79	−0.92 ± 2.58	−2.10 ± 2.68	0.149
CRP (mg/L)	0.005 ± 0.08	0.002 ± 0.04	−0.034 ± 0.12	0.686
Total cholesterol (mg/dL)	−14.89 ± 27.71	−6.25 ± 31.83	−9.26 ± 38.50	0.898
HDL-c (mg/dL)	2.66 ± 7.05	−0.25 ± 10.92	−4.57 ± 12.26	0.402
LDL-c (mg/dL)	0.48 ± 7.38	−5.86 ± 13.19	−4.12 ± 16.84	0.671
Triglycerides (mg/dL)	−2.38 ± 9.23	−3.08 ± 5.51	−3.39 ± 9.39	0.962
Castelli index I	−0.20 ± 1.09	−0.82 ± 1.62	0.20 ± 0.99	0.218
Castelli index II	0.03 ± 0.59	−0.17 ± 0.59	0.13 ± 0.67	0.651

ESPB: extruded sorghum beverage with an added probiotic; ESB: extruded sorghum beverage; CB: control beverage; delta (∆): endpoint—baseline assessment; CRP: C-reactive protein; HDL-c: high-density lipoprotein cholesterol; LDL-c: low-density lipoprotein cholesterol. Castelli index I: total cholesterol/HDL-c; Castelli index II: LDL-c/HDL-c. Different letters mean a statistically significant difference between the groups as determined by ANOVA and a post hoc Newman–Keuls test (*p* < 0.05). The *p*-value in the right column is the *p*-value for the ANOVA. *n* = 10/group.

**Table 4:** Fecal short-chain fatty acid concentrations and fecal pH in adults with overweight and obesity.

Variables	Group			
	ESPB	ESB	CB	*p*-Value
Baseline (mmol/kg)				
Acetic acid	19.53 ± 9.81	19.62 ± 5.80	25.43 ± 7.22	0.171
Propionic acid	13.13 ± 8.08	10.85 ± 4.62	14.92 ± 6.15	0.373
Isobutyric acid	2.06 ± 1.18	2.72 ± 1.03	2.58 ± 1.50	0.494
Butyric acid	16.56 ± 13.06	15.74 ± 9.47	14.48 ± 5.40	0.894
Isovaleric acid	3.46 ± 2.30	4.64 ± 1.96	3.97 ± 2.56	0.537
Valeric acid	2.95 ± 1.37	3.38 ± 1.97	3.40 ± 1.57	0.860
Hexanoic acid	1.49 ± 0.80	0.89 ± 0.63	2.15 ± 1.30	0.222
Total branched-chain SCFAs	5.52 ± 3.46	7.36 ± 0.98	6.55 ± 4.06	0.532
Total SCFAs	59.18 ± 28.53	57.84 ± 23.31	66.93 ± 18.42	0.636
Fecal pH	7.56 ± 0.56	7.27 ± 0.73	7.29 ± 0.59	0.538
Endpoint (mmol/kg)				
Acetic acid	23.66 ± 12.26	18.90 ± 7.44	24.47 ± 12.13	0.472
Propionic acid	16.43 ± 13.12	10.39 ± 5.61	13.18 ± 5.61	0.330
Isobutyric acid	2.14 ± 1.13	1.79 ± 1.01	2.47 ± 1.42	0.455
Butyric acid	18.27 ± 10.15	13.84 ± 11.48	15.68 ± 13.65	0.722
Isovaleric acid	3.77 ± 2.16	3.01 ± 1.77	4.12 ± 2.57	0.517
Valeric acid	3.67 ± 1.85	2.15 ± 1.31	2.95 ± 1.68	0.192
Hexanoic acid	1.66 ± 1.37	0.78 ± 0.56	1.73 ± 1.18	0.396
Total branched-chain SCFAs	5.91 ± 3.26	4.80 ± 2.76	6.59 ± 3.98	0.492
Total SCFAs	69.60 ± 34.10	50.86 ± 25.35	64.60 ± 32.19	0.448
Fecal pH	7.52 ± 0.7	7.17 ± 0.58	7.31 ± 0.59	0.504
Delta (Δ) (mmol/kg)				
Acetic acid	4.13 ± 7.96	−0.72 ± 9.20	−0.96 ± 13.71	0.518
Propionic acid	3.30 ± 7.38	−0.46 ± 6.07	−1.74 ± 7.47	0.287
Isobutyric acid	0.08 ± 1.41	−0.93 ± 1.19	−0.11 ± 1.31	0.209
Butyric acid	1.71 ± 6.61	−1.90 ± 12.13	1.20 ± 14.51	0.765
Isovaleric acid	0.31 ± 1.99	−1.63 ± 2.19	0.15 ± 2.38	0.114
Valeric acid	0.72 ± 1.56 ^a^	−1.23 ± 1.42 ^b^	−0.45 ± 1.62 ^b^	0.042
Hexanoic acid	0.17 ± 1.55	−0.11 ± 0.83	−0.42 ± 1.63	0.865
Total branched-chain SCFAs	0.39 ± 3.39	−2.56 ± 3.37	0.04 ± 3.68	0.142
Total SCFAs	10.42 ± 20.04	−6.98 ± 29.22	−2.33 ± 40.26	0.448
pH fecal	−0.04 ± 0.54	−0.10 ± 0.54	0.02 ± 0.56	0.867

ESPB: extruded sorghum beverage with an added probiotic; ESB: extruded sorghum beverage; CB: control beverage. Results are expressed as means ± standard deviations. Different letters mean statistically significant differences between the groups as determined by ANOVA with a post hoc Newman–Keuls test (*p* < 0.05). The *p*-value in the right column is the *p*-value for the ANOVA. Delta (∆): endpoint—baseline assessment. *n* = 10/group.

## Data Availability

The original contributions presented in the study are included in the article, further inquiries can be directed to the corresponding author.
